# Daily emotional inertia and long-term subjective well-being among people living with HIV

**DOI:** 10.1186/s12955-021-01752-6

**Published:** 2021-03-23

**Authors:** Marcin Rzeszutek, Ewa Gruszczyńska, Ewa Firląg-Burkacka

**Affiliations:** 1 Faculty of Psychology, University of Warsaw, Stawki 5/7, 00-183, Warsaw, Poland; 2 Faculty of Psychology, SWPS University of Social Sciences and Humanities, Chodakowska 19/31, 03-815 Warsaw, Poland; 3 Warsaw’s Hospital of Infectious Diseases, Wolska 37, 01-201 Warsaw, Poland

**Keywords:** Emotional inertia, Affect, Well-being, HIV/AIDS

## Abstract

**Objective:**

The aim of this study was to verify if subjective well-being (SWB) modifies the autoregressive effect of daily emotions and if this emotional inertia predicts long-term changes in SWB among people living with HIV (PLWH).

**Methods:**

The 131 participants had medically confirmed diagnoses of HIV and were undergoing antiretroviral therapy. They assessed their SWB (satisfaction with life, negative affect, positive affect) twice with an interval of one year. They also took part in a five-day online diary study six months from their baseline SWB assessment and reported their daily negative and positive emotions.

**Results:**

Results showed that baseline SWB did not modify the emotional carryover effect from one to another. Additionally, after control for baseline SWB, emotional inertia did not predict SWB one year later. However, such an effect was noted for the mean values of daily reported emotions, indicating their unique predictive power over SWB itself.

**Conclusions:**

This may suggest that emotional inertia does not necessarily provide better information than more straightforward measures of affective functioning.

## Background

There has been a long-standing dispute regarding subjective well-being (SWB) adaptation in response to experiencing various stressful life events [1–4], including the specific case of coping with chronic illness [5]. One of such illness that requires complex psychosocial adjustment and fosters dynamic changes in various components of SWB is HIV/AIDS [6]. Great progress in the treatment of HIV infection has changed HIV/AIDS from a definitely terminal illness to a chronic, but manageable medical condition [7]. Consequently, for an increasing number of PLWH, their health status is still salient, but it is not necessarily the main source of their daily concerns [8, 9]. It has also been found that despite the same source of distress (i.e., HIV infection), stress level in PLWH can vary from day to day, leading to significant individual differences in psychological adjustment in this patient group over time [6]. Some authors link these differences with specific problems in the emotional functioning of many PLWH. This has been referred to as emotion dysregulation expressed as ineffective self-regulation of affective states and difficulties in controlling emotion-driven behaviors on a daily basis [8, 10], being one of the main sources of depression and low quality of life of PLWH in the long term [11]. It has been observed that emotion dysregulation among PLWH may arise from struggling with daily (mainly internalized) HIV-related stigma [8, 12]. In our study, we wanted to examine the above issues from a different perspective that, to the best of our knowledge, has never been applied to this clinical group. Namely, we focused on the relationship between everyday emotional inertia, which may be a proxy of emotional dysregulation, and long-term SWB among the PLWH.

There is an extensive literature on how emotions are related to individuals’ psychological well-being [13–15]. However, it is important to emphasize that the dominant theoretical and methodological assumption of most research in this area is a perspective neglecting within-person dynamics [for a meta-analysis see 16], as emotions are operationalized as single states that can be experimentally turned on and off, or relatively stable individual differences in experiencing particular emotions. This occurs despite the fact that classical authors have underlined that emotions are not static entities but are characterized by inherent dynamics in time [17, 18]. In line with this reasoning, some researchers claim that the above-mentioned static perspective may provide only a limited picture of the emotion–well-being relationship [19]. Therefore, in the last decade, more and more authors postulate a paradigm shift in research on emotions towards emphasizing the temporal dynamics of emotions derived from data collected in natural conditions [20]. For instance, in some studies based on the models of emotions as emergent processes [21], it has been observed that short-term affect dynamics can concurrently and prospectively be related to psychopathological symptoms [22, 23].

One such phenomenon of affect dynamics is emotional inertia. The term was coined by Suls et al. [24] to describe how an individual’s current emotional state may be predicted from his/her previous emotional states. Particularly, the scope of emotional inertia indicates the autocorrelation of emotional trajectories [19, 25]. It was found that high emotional inertia may be a sign of a lack of emotional flexibility and deficits in effective emotion regulation, and thus can predict psychopathology [26]. The studies on emotional inertia, while still very scarce, have left some issues unanswered. First, while the association of emotional inertia with maladjustment has been thoroughly explored [19, 25], the link between emotional inertia and positive aspects of well-being has been highly neglected [26]. Second, to the best of our knowledge, almost all studies on emotional inertia have been conducted in non-clinical settings, and the scarce research on clinical populations has been limited to psychiatric patients only [25].

The strength of emotional inertia is expressed by the autocorrelation between successive emotional states of the same person [19, 25]. It has been found that high emotional inertia may indicate emotional inflexibility and deficits in effective emotion regulation, and thus may predict psychopathology [26]. Studies on emotional inertia, while still very scarce, leave several questions unanswered. First, they mainly concern the relationship between emotional inertia and maladjustment [19, 25], neglecting the positive aspects of well-being [26]. Second, almost all studies on emotional inertia have been conducted in non-clinical settings, and few studies in clinical populations have been limited to psychiatric patients [25].

## Current study

Taking the aforementioned research gaps into consideration, the aim of our study was to examine if in the clinical sample with chronic somatic disease (HIV/AIDS): (a) SWB modifies the autoregressive effect of daily emotions, replicating the effect reported in other samples; and (b) daily emotional inertia predicts SWB after control for baseline SWB and mean values of daily reported emotions. We wanted to examine whether SWB changes across one year can be predicted from the daily emotional carryover effect. Additionally, we assumed that the inertia effect is stronger for affective than cognitive components of SWB and stronger for negative emotion inertia than positive emotion inertia.

## Method

### Participants

The sample consisted of 131 participants with confirmed diagnoses of HIV infection. The majority were men (85.5%), with a university degree (64%) and stable employment (75%). Fifty percent of participants declared that they were single. The mean age was 39.3 ± 10.3 years, with an average duration of HIV infection of 7.2 ± 6.2 years (ranging from 1 to 30 years). All the participants had been on antiretroviral treatment for at least one year (the mean was 5.8 ± 6.3 years), and 14% were diagnosed with AIDS. The average CD4 count was 586.199 ± 264.282.

The participants were recruited during a control visit to an outpatient clinic where they were receiving antiretroviral treatment. The additional inclusion criteria included a lack of illness-related cognitive disorders and no current diagnosis of substance dependence. For practical reasons, access to the Internet was also required. Participation was voluntary and participants were not remunerated.

Out of 153 participants available for the study, 22 provided no diary data and were excluded from further analysis. Additionally, 96 persons took part in the final well-being assessment, representing a 37% dropout rate. However, it could be regarded as missing at random (Little’s MCAR test: χ^2^ = 9.72, df = 7, *p* = 0.205). Thus, all the analyses were conducted for N = 131, using all the available data.

### Procedure

The procedure included traditional longitudinal design with a burst of intensive longitudinal design. The participants took part in two measurements of subjective well-being, separated by 12 months. The same group completed online daily diary measures of their end-of-day positive and negative emotions during five consecutive days, from Monday till Friday, starting six months after the first measurement of well-being.

The study protocol was approved by the local ethics committee, and informed consent was obtained from all the participants. The longitudinal part of the study was done with a paper-and-pencil approach; the participants were contacted in person by the research assistants at an outpatient clinic during their scheduled control visits. The diary part of the study was conducted online, with time-stamped questionnaires sent via e-mail each evening as hyperlinks, accessible from Internet-connected PCs, smartphones, and tablets. Access to the online measurement was restricted to a limited time daily, and when the questionnaires were sent back, there was no possibility to look at or review previous answers.

### Measures

Subjective well-being was assessed with two questionnaires to measure its cognitive and affective components: the Satisfaction with Life Scale (SWLS) [27] and the Positive and Negative Affect Schedule (PANAS-X) [28]. The SWLS consists of five items describing personal satisfaction with one’s life as a whole. The participants evaluated each item on a 7-point scale ranging from 1 (*strongly disagree)* to 7 (*strongly agree)*. Therefore, a higher total score on this scale indicates a higher level of satisfaction with life. Cronbach’s alpha coefficient in the studied sample was 0.87.

Positive and negative affect (PA, NA) refer to the affective component of subjective well-being. This was evaluated using 20 descriptions of feelings and emotions: 10 for positive affect and 10 for negative affect from the PANAS-X by Watson and Clark [28]. The participants rated their generally experienced affective states on a 5-point response scale from 1 (*not at all*) to 5 (*strongly*). Cronbach’s alpha coefficients obtained in this study were 0.91 for the positive affect scale and 0.89 for the negative affect scale.

Daily positive and negative emotions were also assessed using the PANAS-X, but with a shorter version that included 12 items: six for positive emotions (relaxed, excited, energetic, calm, glad, satisfied) and six for negative emotions (angry, concerned, unhappy, worried, tired, discouraged). The participants evaluated how they felt right now (that is, at the end of each day) and provided their answers on a 5-point scale from 1 (*not at all*) to 5 (*strongly*). The multilevel reliability (omega coefficient) is 0.81 and 0.80 for within-person level for negative and positive emotions, respectively, and 0.94 for between-person level for both valences.

### Data analysis

The analysis was based on a multilevel autoregressive model in which data on emotional inertia was obtained from a two-level model, with days nested in persons, leading to 655 measurement points [29]. Each model contained a random intercept and a random slope with latent person-mean centering [30, 31]. Specifically, at the within-person level of this model, the degree to which emotions reported at Day *i* were predicted by emotions reported at Day *i* − 1 was evaluated*.* At the between-person level of this model, person-specific intercept and slope values were estimated. The intercept represents individual differences in the average level of emotions reported during the diary study, whereas slope represents individual differences in inertia (i.e., in day-to-day autocorrelation of emotions). Random intercept and random slope were allowed to covary across participants. In this analysis, only diary data were used.

In the next stage of the between-person part of the model, the cross-level interaction was added to examine the relationship between inertia and baseline well-being indicators (SWL, NA and PA, all centered around grand mean). A separate model was tested for each indicator.

Finally, linear regression was performed with well-being indicators at Time 2 as dependent variables and random intercept and random slope as explanatory variables after control for baseline well-being and selected demographic variables (sex, age, education, relationship status and employment status) and clinical variables (duration of HIV infection, duration of antiretroviral treatment, CD4 count, and AIDS stage). With Bayesian estimation, and under the assumption of a missing at random pattern, all the available data in a way optimal for modeling were used [31]. The analyses were conducted with IBM SPSS version 25 and Mplus version 8.2.

## Results

### Descriptive statistics and preliminary analysis

Table 1 presents descriptive statistics of the variables used in the study. For negative and positive emotions, they represent values aggregated across persons and days. Subjective well-being indicators at Time 2 were related only to some demographic and clinical variables. For SWL significant correlate was education (*β* = 0.32, *p* < 0.001), whereas for PA relationship status (*β* = 0.34, *p* < 0.00) and CD4 count (*β* = −0.25, *p* = 0.008). No significant correlates for NA at Time 2 were found. Thus, in the regression analysis models, only selected control variables are included, depending on the explained variable.

**Table 1:** Descriptive statistics for the study variables

Variable	M	SD	Min–Max	Skewness	Kurtosis
**Daily emotions**					
NE	2.04	0.84	1–5	0.98	0.32
PE	2.91	0.89	1–5	− 0.08	− 0.55
**Subjective Well-being**					
SWL_1	20.87	6.38	5–35	− 0.30	− 0.61
SWL_2	19.34	5.79	5–35	0.20	− 0.06
NA_1	2.19	0.88	1–4.5	0.69	− 0.38
NA_2	2.10	0.74	1–3.9	0.55	− 0.88
PA_1	3.36	0.73	1–4.7	− 0.43	− 0.07
PA_2	3.30	0.72	1.6–4.6	0.14	− 0.61

M—mean; SD—standard deviation; NE, PE—Daily negative and positive emotions, respectively; SWL—Satisfaction with life; NA, PA—Negative and Positive affect, respectively; subscripts 1 and 2 denote first and second measurement

### Average emotional inertia and its relationship with baseline subjective well-being

As shown in Table 2, the average emotional inertia for NA and PA is positive and significant, which suggests an expected carryover effect from one day to the next. Specifically, a higher level of negative emotions the day before was associated with a higher level of this emotion on the following day. The same pattern was observed for positive emotions. The explained variance expressed by within-level R-square averaged across clusters was 25% and 22% for NA and PA, respectively.

**Table 2:** Results of multilevel autoregressive models estimating emotional inertia and its associations with baseline level of subjective well-being model results

Model	Daily negative emotions			Daily positive emotions		
	Est	95%CI		Est	95%CI	
		LL	UL		LL	UL
**Autoregressive only**						
Raw						
mean	2.01***	1.79	2.14	2.90***	2.75	3.06
inertia	.45***	.34	.55	.38***	.24	.52
Standardized						
mean	1.74***	0.95	3.62	1.26***	0.66	2.21
inertia	.44***	.35	.53	.38***	.23	.51
**Satisfaction with life**						
mean	− .44***	− .72	− .18	.54***	.36	.93
inertia	.03	− .25	.32	.08	− .19	.38
**Negative affect**						
mean	.23	− .04	.48	− .21	− .46	.01
inertia	.16	− .16	.51	.01	− .33	.24
**Positive affect**						
mean	− .31***	− .61	− .03	.48***	.29	.84
inertia	− .26	− .59	.00	− .22	− .51	.02

Standardized results are within-level standardized estimates averaged over clusters. All the results provided for well-being indicators are standardized. ***p < .001

Additionally, subjective well-being did not modify this carryover effect. Satisfaction with life and positive affect were significantly related only to random intercept, indicating that people who had a higher SWL and PA also reported a higher average level of positive emotions and lower average level of negative emotions during the diary part of the study.

### Emotional inertia as predictor of subjective well-being

Table 3 shows the results of regression models longitudinally predicting subjective well-being from emotional inertia after control for baseline well-being, average level of emotions reported during daily dairy and selected sociodemographic and clinical variables. For all the subjective well-being indicators, neither PA nor NA inertia predicts well-being change within one year among PLWH. In each model, the baseline level of well-being is significantly associated with the outcome. Additionally, an average PA reported during diary assessment is a positive predictor of SWL and PA and a negative predictor of NA, which means that it uniquely predicts well-being above and beyond its baseline values. For an average daily NA, this is the case only for NA.

**Table 3:** Results of linear regression models predicting subjective well-being one year later from emotional inertia after control for well-being baseline level

Model	Satisfaction with life			Negative affect			Positive affect		
	Est	95% CI		Est	95% CI		Est	95% CI	
		LL	UL		LL	UL		LL	UL
**Daily negative emotions**									
Baseline	.40***	.28	.50	.18**	.05	.31	.41***	.29	.51
NE mean	− .45	− .79	.02	.65**	.14	.95	− .30	− .75	.14
NE inertia	.01	− .49	.49	− .004	− .49	.43	− .03	− .48	.48
*R*^2^	.47***	.22	.92	.52***	.10	.94	.40***	.22	.88
**Daily positive emotions**									
Baseline	.35***	.22	.47	.19***	.06	.31	.37***	.24	.48
PE mean	.57***	.22	.88	− .43*	− .94	− .01	.55***	.10	.86
PE inertia	− .08	− .44	.26	.13	− .27	.52	− .10	− .50	.24
*R*^2^	.54***	.26	.94	.29***	.06	.94	.56***	.26	.95

All the provided results are the within-level standardized estimates averaged over clusters. For Satisfaction with life and Daily negative emotions model: education = .20, 95% CI (.08, .31), *p* < .001. For Satisfaction with life and Daily positive emotions model: education = .19, 95% CI (.07; .31), *p* < .001. For Positive affect and Daily negative emotions model: relationship status = .20, 95% CI (.09, .31), *p* < .001; CD4 count = − .17, 95% CI (− .27, − .06), *p* < .001. For Positive affect and Daily positive emotions model: relationship status = .20, 95% CI (.09, .32), *p* < .001; CD4 count = − .14, 95% CI (− .25, − .04), *p* = .005. Education was coded 1—*having a university degree*, 0—*not having a university degree*. Relationship status was coded 1—*being in a stable intimate relationship*, 0—*being single*

***p < .001, **p < .01, *p < .05

## Discussion

The results of our study were inconsistent with our research hypothesis. We observed that SWB does not modify daily emotional inertia and that daily emotional inertia does not predict one year changes in SWB with regard to any of its components (SWL, NA, and PA). As far as these separate SWB elements are concerned, we found an interesting pattern illustrating that only mean values of collected daily PE and NE added significantly to prediction of long-term SWL (only mean values of daily PA), long-term PA (only mean values of daily PA), and long-term NA (both daily PA and NA), after control for baseline level of each of these components.

In trying to interpret the aforementioned, unexpected null result regarding the emotional inertia, it is vital to underline that contemporary research on this phenomenon focused on its link predominantly with negative measures of well-being or psychopathology [for meta-analysis, see 16], which may lead to bias in extrapolating these findings on positive indicators of well-being [26]. It can therefore be assumed that, in the light of current knowledge emotional inertia should be treated less as a predictor of broadly operationalized well-being and more as merely a correlate of maladjustment or psychopathology only [26, 32]. Lastly, the null result obtained in our study could also stem from the fact that we focused on emotional inertia as a predictor of SWB change and not on a cross-sectional relationship between these constructs, a design that dominates in emotional inertia research [16]. However, among PLWH, SWB did not moderate autoregressive effect of both valences of daily emotions, thus this finding was also not replicated.

In this context, it is worthwhile to mention that some authors are generally more skeptical about the utility of emotional inertia and other modern conceptualizations of emotional regulation expressed by complex dynamic measures (e.g., emotion variability, emotional instability) in predicting psychological well-being or psychopathology [33, 34]. Specifically, a recent meta-analysis conducted by Dejonckheere et al. [34] showed that dynamic affect measures entail very limited added value over mean levels of positive and negative affect in the process of predicting individual differences in well-being (i.e., life satisfaction, depressive symptoms, and borderline symptoms). When there is a control for the mean level of PA and NA, which was the case in our study, a lack of unique explanatory power of these dynamic constructs is likely to be observed. Thus, the effects of these dynamic measures of affect should always be adjusted for an average emotional intensity [33].

The obtained results should also be discussed in the context of the specificity of the studied sample. Distress associated with a serious chronic health condition affects many areas of daily functioning, but long-term patterns of affective adaptation to such conditions tend to follow the hedonic treadmill model [4], i.e., "stability despite loss" [35]. It was found that despite daily emotion dysregulation due to HIV stigma [8, 11] PLWH also display remarkably stable PA and NA many years after HIV diagnosis, which is not associated with HIV-related biomarkers [36–38].

Thus, dysregulation, even if present, did not necessarily lead to long-term changes in well-being, which is similar to our results.

However, one may assume that among PLWH some atypical values of emotional inertia could be found, but it seems unlikely. On average, the inertia level and direction tend to be similar to values already reported in other samples [16]. Moreover, our participants showed a substantial variability in this regard; there were some for whom high emotional carryover from one day to the next one could be found, and others for whom this value was close to zero. Thus, there is no reason to consider that the obtained results reflect highly specific processes not observable in other groups.

To sum up, in the studied sample of PLWH there was a significant inertia for both positive and negative emotions. However, it did not depend on the baseline level of well-being, nor was it a predictor of its change, both in terms of satisfaction with life and long-term affective functioning.

## Strengths and limitations

Our study has several unique strengths: it is a theory-driven, longitudinal assessment of study variables from two temporal modes (i.e., daily fluctuations and long-term changes). Also, taking into account the complexity of the study design (and especially the clinical sample of PLWH, which had never previously been investigated in the context of emotional inertia), the number of participants can be regarded as sufficient. In terms of limitations, the daily diaries consisted of only a few days; this might affect the validity of the inertia indicator. Additionally, we probably could not have avoided a selection bias typical of extensive study designs, with significant burden over the participants, as our sample consisted of highly functional PLWH with well-controlled HIV infection. Thus, it is also important to consider the broader socio-cultural context relevant to research on the well-being of PLWH [38]. In short, it concerns mainly illness perception, minority stress, and stigmatization at both individual and societal level, which are associated with objective and subjective determinants of access to treatment [39]. In Poland most of the funding from the National HIV Prevention and AIDS Control Program [40] goes to treatment rather than prevention and education, and access to mental health care for the HIV/AIDS population is still very limited. Therefore, it would be worthwhile additionally control the results for depression symptoms. Nonetheless, every person diagnosed with HIV has guaranteed access to free antiretroviral treatment, compliant with the current World Health Organization guidelines. In this sense, the study sample is fully homogeneous, although may differ significantly from samples with more limited access to treatment.

## Conclusions

The results of our study show that more caution is needed in implementing novel theoretical constructs regarding emotion regulation and related complex and dynamic measures. As promising as they can be, the uniqueness of these constructs’ explanatory power over more straightforward emotion indicators is still waiting to be proven. Until then, there is a risk of multiplying redundant findings, especially when there is a publication selection bias in favor of positive results. This bias may create an illusion of progress in emotion research, but in fact it is *reinventing the wheel* [37]. Overall, it seems that while emotional inertia is somehow related to dysfunctional patterns of affect in psychopathology [15], it does not exert a significant effect within the more normative range of affective functioning [36], including among PLWH [41]. These latter results should be taken into an account in developing interventions to improve the quality of life of PLWH, especially if the targeted mediating factor is an effective emotion regulation. For example, future studies should concentrate on the exploration of emotional inertia in the context of daily HIV/AIDS stigma. If emotional inertia does not predict PLWH’s well-being, the open question is whether it could be responsible for the mechanism of stigma-related emotion dysregulation in this patient group on a daily basis [8].

## Data Availability

The datasets used and/or analysed during the current study available from the corresponding author on reasonable request.

## Abbreviations

PLWH: People living with HIV
SWB: Psychological well-being
PA: Positive affect
NA: Negative affect
